# A Fuzzy-Decision Based Approach for Composite Event Detection in Wireless Sensor Networks

**DOI:** 10.1155/2014/816892

**Published:** 2014-07-21

**Authors:** Shukui Zhang, Hao Chen, Qiaoming Zhu, Juncheng Jia

**Affiliations:** ^1^School of Computer Science and Technology, Soochow University, Suzhou 215006, China; ^2^State Key Laboratory for Novel Software Technology, Nanjing University, Nanjing 210093, China

## Abstract

The event detection is one of the fundamental researches in wireless sensor networks (WSNs). Due to the consideration of various properties that reflect events status, the Composite event is more consistent with the objective world. Thus, the research of the Composite event becomes more realistic. In this paper, we analyze the characteristics of the Composite event; then we propose a criterion to determine the area of the Composite event and put forward a dominating set based network topology construction algorithm under random deployment. For the unreliability of partial data in detection process and fuzziness of the event definitions in nature, we propose a cluster-based two-dimensional *τ*-GAS algorithm and fuzzy-decision based composite event decision mechanism. In the case that the sensory data of most nodes are normal, the two-dimensional *τ*-GAS algorithm can filter the fault node data effectively and reduce the influence of erroneous data on the event determination. The Composite event judgment mechanism which is based on fuzzy-decision holds the superiority of the fuzzy-logic based algorithm; moreover, it does not need the support of a huge rule base and its computational complexity is small. Compared to CollECT algorithm and CDS algorithm, this algorithm improves the detection accuracy and reduces the traffic.

## 1. Introduction

In the event-driven wireless sensor networks (EWSNs) [1], a large number of sensor nodes deployed in monitoring area detect interested physical phenomena (such as forest fire prevention) in the environment. Event is the state changes in the real world [2]. Most previous studies of event detection just consider simple events. The literature [3] proposes distributed Bayesian algorithm for the area detection of the fault-tolerant events; this algorithm determines whether an event has occurred in its self-position through exchanging information with surrounding nodes. The literature [4] shows three ways to determine whether the nodes are at the boundary of the event; these three methods are based on statistics, image processing algorithm, and classification algorithm, respectively. The position of the node and judgment will be sent to the base station, and then the base station judges the area of the event. The literature [5] presents fault-tolerant events area detection mechanism by combining with space-time correlation and threshold. The literature [6] analyzes the simple event detection delay; only a kind of sensor is needed in the network in the judgment of the simple event. However, most events need to detect various properties of the environment, if sensors that are needed to detect the Composite event can be integrated on a node, and then Composite event can be regarded as a simple event [7].

In recent years, heterogeneous wireless sensor network [8] (WHSN) consisted of different types of nodes has appeared; different types of nodes in the network integrate into different sensors. For example, there may be three kinds of nodes in the wireless sensor network (WSN) to detect fire; temperature, smoke concentration, and light intensity sensors are integrated into each node separately.

Wireless heterogeneous sensor network (WHSN) is suitable for Composite event detection [9], and there are two ways to deploy the nodes: nonrandom deployment and random deployment. For these two kinds of deployment, there are many literatures that have discussed the distributed algorithms. In the literature [10, 11], the monitoring area is divided into multiple virtual grids with the same size, and each grid has a gateway node. The activated nodes provide the gateway node with environmental information, and then the gateway node judges whether the Composite event has occurred. Such algorithms assume that the sensing range of the node can cover the whole grid, and there are sufficient nodes in the girds to determine whether the Composite event occurs. The literature [12] studies the coverage of Composite events on the basis of dividing the grid. Whether Composite event occurs can be determined through exchanging information with neighboring under random deployment. According to the literature [13], CollECT algorithm is proposed for Composite event detection and tracking problems. Since CollECT algorithm requires that each node be active, therefore the traffic of this algorithm will be heavy when the event occurs. The literature [14] shows a method which is based on connected dominating set (CDS) for Composite events detection, but the accuracy is not high. Compared with CollECT algorithm, CDS algorithm detects the Composite events by the sleep scheduling mechanism, and it reduces the network energy consumption effectively. In addition, the algorithm only needs active nodes to know their own locations; thus it reduces the position overhead.

A reliable routing mechanism is needed to ensure that the event information can be transmitted to the base station successfully after the events are detected. Reliable routing mechanism often establishes multiple paths between the source node and the base station, and event information can be sent to the base station as long as at least one path is valid. The literatures [15, 16] establish multiple disjoint paths between the source node and the base station to ensure the reliability of data transmission. The literature [17] proposes a SSR protocol, which requires data packets to consider the situation of surrounding nodes and choose the next-hop node properly in every transmission. Cut point will appear in the network when the node fails. When the cut point fails, the event information could not be transmitted to the base station, if all the paths between the source node and the base station pass the cut point. Therefore, it is necessary to fix the cut points. The literature [18] summarizes the existing cut point predicting algorithm and analyzes its performance. The literature [19] discusses how to repair the cut points in the case that there are mobile nodes in the network. When the link that reaches the next hop in the routing table fails, flooding [20] can be used to set a new route to forward the event information.

The above literatures have researched the network topology and routing. They all use threshold-based determination mechanisms; that is, when a node senses that a property's value is more than a given threshold, it considers that event related to the property occurs. In the literature [4], each node uses this strategy to determine whether the simple event occurs, then exchanges information with neighbor nodes, and finally determines their own verdict to ensure the boundaries of simple events. In the literatures [2, 10], the Composite event detection is achieved by the following process: first of all, whether simple events corresponding to different sensors reach a given threshold, Boolean calculation is operated on the result of these events to get the result of the determined events. However, it is not reasonable to determine the events in nature through the threshold mechanism. Since data around the threshold may affect the test result, and as a result of many other factors, part of data produced by sensor in the sensing process may be wrong.

Because the definition of the event in the realistic environment is uncertain, and fuzzy-logic based Composite events detection is raised in the literature [21]. Fuzzy logic has had some applications in the wireless sensor network (WSN); literatures [22–24] use fuzzy logic method to select cluster heads, literatures [25, 26] use it to solve the safety problem in wireless sensor network, literature [27] utilizes it in data fusion, and literature [28] imposes it to design routing algorithms. Decision mechanism based on fuzzy logic divides simple events into different levels firstly, calculates the membership of the detected attribute value to each level with membership function, then calculates each rule in the rule base, and finally obtains result with anti-fuzzy.

Objectively, Composite events decision mechanism which is based on fuzzy-logic resolves many unreasonable problems resulting from using threshold value to determine events, but as the decision mechanism needs huge rule base, and rule base will grow exponentially with the number of attributes to be detected increase, thus, it puts forward higher requirements for the sensor node's storage capacity.

In this paper, the proposed fuzzy-decision based Composite event detection algorithm (FL-CED) consists of three parts: first of all, building network topology based on connected dominating sets; the dominant point is local decision node; then using space-time correlation to cluster the perceived value of the same attribute, screening out the correct data, and identifying the fault node; finally, determining Composite events with decision-making mechanism based on fuzzy-decision. The construction of the network topology based on connected dominating set forms the spanning tree which regards the control point as the decision point, and clustering algorithm ensures the correctness of the data involved in determining. Because decision-making mechanism based on fuzzy-decision does not need the support of huge rule base, it is more practical than the decision-making mechanism based on fuzzy logic.

## 2. Network Model

Let *N* sensor nodes randomly deploy in the detection area *A*, so that the monitoring area is substantially covered and each node is integrated by one or more sensors. Different sensors have different sensing ranges and thus have a different perception and the communication radius is adjustable. Maximum communication radius is described as *R*_*C*_. The network consisting of nodes is connected; each node with a unique ID determines its own position by localization algorithm [29, 30] or GPS [31].

Generally, the signal energy transmitted from sensor node is decreasing as the distance from the signal source is increasing. Assuming that *d*_*i*_ is the distance between node *i* and the event source, and *S* is the signal energy of the detection node, the lost energy is *s*_*i*_ = *S* × *w*(*d*_*i*_), and *w*(0) = 1, *w*(*∞*) = 1, and *w*(*x*) = *θ*(*x* ^−*k*^), where *w*(*x*) is the signal attenuation function, and it is subject to climatic and other environmental conditions; *k* is loss index of the signal transmission, and its range is from 2.0 to 5.0. In this paper, the signal attenuation function [32] is as follows:

(1) $$\begin{array}{c} w\left(x\right)=\frac{1}{1+x^{k}}. \end{array}$$

Errors of sensing data of the sensor mainly come from the hardware or the random ambient noise. *H*_0_ and *H*_1_ denote “event does not occur” and “event occurs,” respectively. *x*_*i*_ represents the measured data of node *i*: *H*_0_: *x*_*i*_ = *n*_*i*_; *H*_1_: *x*_*i*_ = *s*_*i*_ + *n*_*i*_. *n*_*i*_ is the noise energy received by the node *i*. Let noise *n*_*i*_ be distributed normally at each node *i*, *n*_*i*_ ~ *N*(*μ*,*σ* ^2^), where *μ* and *σ* are the mean and variance, respectively. The noise {(*n*_*i*_∣∀ *i*)} is independent of the sensor and obeys normal distribution. The sensing data of the detection node is a normal distribution: *x*_*i*_∣*H*_1_ ~ *N*  (*s*_*i*_ + *μ*, *σ* ^2^). Due to the independence of the noise, the energy of the received signal of different nodes is different, but the detected value {*x*_*i*_∣∀ *i*, *H*_1_} of the sensor nodes is independent of the space. The peak value of the signal to noise ratio (SNR) is defined as  *δ* = *s*/*σ* to quantify the level of the noise. The signal attenuation and noise model that obeys independent normal distribution has been widely applied in multisensor node signal detection and has been shown [32]. In fact, noise transmission is an approximate normal distribution. The model (i.e., *s*, *w*(*x*), *μ*, and *σ* ^2^) can be used to estimate the sensing data collected by the WSNs.

## 3. Definitions

**Definition 1 (perceived neighbors).**

*V*  is the set of all nodes, *u*, *v* ∈ *V*; let *v* be a sensor *C*_*i*_ with a sensing property *A*_*i*_. The perception radius of *C*_*i*_ is *R*_*Si*_. If *d*(*u*, *v*) ≤ *R*_*Si*_, (*d*(*u*, *v*) represents the Euler distance between *u* and *v*), then *v* is the *A*_*i*_-aware neighbor of *u*. *v* is the *A*_*i*_-aware neighbor of *u*, which means *v* can sense attribute *A*_*i*_ of *u*.

**Definition 2 (active sensing neighbors).**

The nodes that are not greater than *R*_*si*_/*η* (*η* is an integer greater than 1) distance from node *u* are called the effective *R*_*si*_ perception neighbor of *u*, and the set of all the points satisfying the properties is denoted by *N*_*i*_(*u*) (the selection of *η* will be discussed in Section 8.3).

**Definition 3 (simple event).**

The event occurred can be determined by detecting an attribute of the environment, while the event is called a simple event, denoted as

(2) $$\begin{array}{c} e=\{\begin{array}{cc} H_{0},\;P\left(x\right)=\text{false} & \\ H_{1},\;P\left(x\right)=\text{true,} & \end{array} \end{array}$$

where *e* means a simple event and *x* means the corresponding environment attribute value of event *e*. When its value is true, it indicates that the event occurred. When the value is false, it indicates that the event did not occur.

**Definition 4 (composite event).**

Only detecting multiple attributes of the environment can determine whether an event occurs; then the event is called Composite event, denoted as

(3) $$\begin{array}{c} E=\{\begin{array}{cc} H_{0},\;P\left(x_{1},x_{2},\ldots ,x_{m}\right)=\text{false} & \\ H_{1},\;P\left(x_{1},x_{2},\ldots ,x_{m}\right)=\text{true,} & \end{array} \end{array}$$

where *E* represents the Composite event, *x*_1_, *x*_2_,…, *x*_*m*_ represent *m* kinds of attributes values of the environment, and *P*(*x*_1_, *x*_2_,…, *x*_*m*_) is the decision function, whose meaning is the same with the simple event.

**Definition 5 (event area).**

If detecting events (simple event or Composite event) in any position of area *A*, the results are all *H*_1_, then area *A* is called the event area.

In the boundary of the event area, since attribute values of the environment do not vary, the environmental attribute values detected by the node can approximately reflect the state of the attribute in the node sensing range. Because of harsh environmental conditions, nodes are likely to be damaged. Then we can deploy many nodes of the same type to detect the region. Based on the* k-watching* nature of the event in literature [10], we propose a regional* k-watching* concept.

**Definition 6 (*k*-watched area).**

If all attributes *A*_1_, *A*_2_,…, *A*_*m*_ of the environment are detected by the node integrated by *K* sensors *C*_1_, *C*_2_,…, *C*_*m*_, respectively, the region is* k*-watched, and the region also meets the* k*-watching nature. If *N*_*i*_(*u*)  (1 ≤ *i* ≤ *m*) of node *u* in the monitoring area has *K* nodes integrated *C*_*i*_, respectively, then *u* is said to be* k*-watching decision node, referred to as a decision node, *R*_*d*_ = (1 − 1/*η*)min⁡(*R*_*s*1_, *R*_*s*2_,…, *R*_*sm*_) is called the* k*-watching policy area, referred to as decision-making scope.

**Definition 7 (detecting voids).**

If a node is not the decision node, and it is not in a decision-making scope of a decision node, then the events surrounding the node could not be detected; the location of the node is called a detection void.

**Definition 8 (clustering [33]).**

Let *X* be a data set, *X* = {*x*_1_, *x*_2_,…, *x*_*k*_}; *x*_*i*_ denotes properties of object *i*. An* m*-cluster of *X* is donated as *R*; then *X* is divided into *m* classes *C*_1_, *C*_2_,…, *C*_*m*_ by *R* and satisfies the following three conditions:

1. *C*_*i*_ ≠ *∅*, *i* = 1,…, *m*;
2. ∪_*i*=1_^*m*^ *C*_*i*_ = *X*;
3. *C*_*i*_∩*C*_*j*_ = *∅*, *i* ≠ *j*, *i*, *j* = 1,…, *m*.

**Definition 9 (membership function).**

An arbitrary mapping from the domain *X* to the closed interval [0,1]: $u_{\tilde{A}}:X\to \left[0,1\right]$, for any *x* ∈ *X*, $x\to u_{\tilde{A}}(x)$, $u_{\tilde{A}}(x)\in [0,1]$; then $\tilde{A}$ is called a fuzzy subset of *X*, $u_{\tilde{A}}$ is called membership function of *x*, and $\mu_{\tilde{A}}(x)$ is called the membership of *x* to fuzzy set $\tilde{A}$, denoted as

(4) $$\begin{array}{c} \tilde{A}=\left\{\left(x,u_{\tilde{A}}\left(x\right)\right)\;|\;x\in X\right\}. \end{array}$$

Given a limited domain *X* = {*x*_1_, *x*_2_,…, *x*_*m*_}, its fuzzy subset $\tilde{A}$ can be denoted as Chad notation:

(5) $$\begin{array}{c} \tilde{A}=\frac{u_{\tilde{A}}\left(x_{1}\right)}{x_{1}}+\frac{u_{\tilde{A}}\left(x_{2}\right)}{x_{2}}+\cdots +\frac{u_{\tilde{A}}\left(x_{i}\right)}{x_{i}}+\cdots +\frac{u_{\tilde{A}}\left(x_{n}\right)}{x_{n}}, \end{array}$$

where *x*_*i*_ ∈ *X*  (*i* = 1,2,…, *n*) is the element of the domain, $\mu_{\tilde{A}}\left(x_{i}\right)$ is the membership function of *Xi* to $\tilde{A}$, $0\leq \mu_{\tilde{A}}\left(x_{i}\right)\leq 1$, the formula represents a fuzzy set of *n* elements, and “+” is sign for Chad mark.

## 4. Criteria

Assuming that detecting Composite event *E* needs to detect environmental attributes *A*_1_, *A*_2_,…, *A*_*m*_, these properties are detected by the integrated sensor *C*_1_, *C*_2_,…, *C*_*m*_, corresponding perception range is *R*_*s*1_, *R*_*s*2_,…, *R*_*sm*_, respectively, and *x*_1_, *x*_2_,…, *x*_*m*_ are the values of corresponding attribute. The node that detects *P*(*x*_*i*_) = *true* sends the message (attribute, value) to the local decision node, and the concrete properties values are not contained in the message, which only contains 0 or 1; 1 shows that *P*(*x*_*i*_) = *true* and can reduce the length of data packets. Local decision node operates after receiving packets and judges whether event happens within the decision scope.

*Criteria*. For any node *u* in the network, *N*_1_(*u*), *N*_2_(*u*),…, *N*_*m*_(*u*) have the node with integrated sensors *C*_1_, *C*_2_,…, *C*_*m*_, and their readout are *x*_1_, *x*_2_,…, *x*_*m*_; if *P*(*x*_1_, *x*_2_,…, *x*_*m*_) = *true*, Composite event could happen in the circular area, which regards *u* as the center and *R*_*d*_ = (1 − 1/*η*)min⁡(*R*_*s*1_, *R*_*s*2_,…, *R*_*sm*_) as radius; the set of all nodes within decisions scope of *u* is denoted as *N*_*D*_(*u*).

**Theorem 1 .**

For any node *u* in the network, one can choose an node integrated sensor *C*_1_, *C*_2_,…, *C*_*m*_ from *N*_1_(*u*), *N*_2_(*u*),…, *N*_*m*_(*u*), a collection constituted of these nodes is recorded as {*v*_1_, *v*_2_,…, *v*_*m*_} (elements can be repeated), and if there is such a {*v*_1_, *v*_2_,…, *v*_*m*_}, {*v*_1_, *v*_2_,…, *v*_*m*_} can sense any environment attributes of any position in the decision scope of *u*.

**Proof**

**Figure 1:**
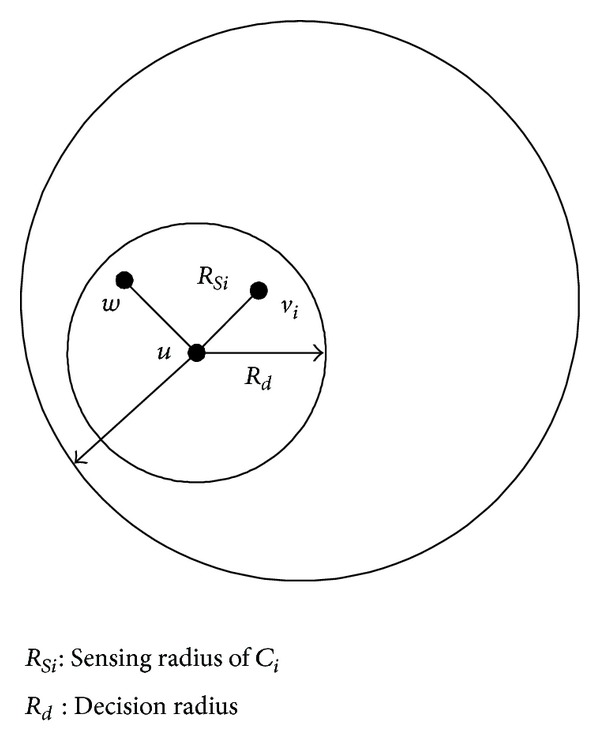
Decision making range diagram.

Take a *w* within the decisions scope of *u* and decision radius of *u* is *R*_*d*_. Assuming that the distance *d*(*u*, *v*) between *u* and *v* is less than *R*_*d*_ and the distance *d*(*u*, *v*) between *u* and node *v*_*i*_ integrated *C*_*i*_ is less than *R*_*Si*_/*η*, see Figure 1 then

(6) d(w,vi)≤d(w,u)+d(u,vi)≤Rd+RSiη≤(1−1η)RSi+RSiη=RSi.

Thus, environment attributes *A*_*i*_ of *w* can be tested by *v*_*i*_. Since *w* and *i* are arbitrary, {*v*_1_, *v*_2_,…, *v*_*m*_} can sense any environment attribute within any position of decision scope of *u*.

Theorem 1 shows that as long as selected part of decision nodes is in the monitoring area and other nodes are within the scope of their decisions, then we can determine the status of the incident in this monitoring area by testing the environment with these decision nodes. However, attribute values received by local decision node not necessarily reflect changes of the environment correctly. Decision mechanism proposed in this paper is divided into two parts: first, the local decision nodes process the detecting data with clustering algorithm and use the processed data to make fuzzy decision and get the final conclusion.

## 5. Construction of the Network Topology

Abstracting the network into a graph *G* = (*V*, *E*), *G*′ = (*V*′, *E*′) and *G*_*D*_ = (*V*, *E*_*D*_), *V* represents the set of all the nodes in the network, *E* represents the set consisted of neighbor node pairs communicating with each other in *V*, and *V*′ = *V* + {sink}, where sink is the base station. *E*′ represents the set consisted of neighbor node pairs communicating with each other in *V*′; *E*_*D*_ is the set consisting of node pairs whose Euler distance is not more than *R*_*d*_. The CETC algorithm proposed in this paper regards dominating sets as the topology of the event detection and selects a subset of the graph *G* as *V*_*D*_, where the point is the local decision node. The choice of *V*_*D*_ should meet the following demands.

1. Nodes in *V*_*D*_ are decision nodes.
2. *V*_*D*_ is a dominating set of *G*_*D*_ = (*V*, *E*_*D*_); domination relationship is the decision making.
3. Use a distributed algorithm, each node can decide whether to be a point of *V*_*D*_ in local scope.
4. Algorithm has lower time complexity and message exchange complexity.
5. Nodes in *V*_*D*_ have more residual energy than other nodes.
6. The number of points in *V*_*D*_ should be less as possible and these points are scattered in space.

Based on the goal above, the process of building the topology structure of the network is divided into two steps: selecting *V*_*D*_ and *V*_*s*_. Selecting *V*_*D*_ is selecting a dominating set of *G*_*D*_. Selecting *V*_*s*_ is selecting part of the nodes in *V*, providing the nodes in *V*_*D*_ with attributes that they cannot sense, and making the decision range of the node in the *V*_*D*_ satisfy the* k*-watching nature. Initially, there are four types of nodes in the network.

1. Sleep node (SLEEP): node inactive.
2. Local decision node (DECIDE): nodes collecting the information of peripheral nodes and determining whether the Composite events happen within *R*_*d*_.
3. Sensor nodes (SENSE): perceiving attribute values of the environment and sending them to the local decision node.
4. Connect nodes (CONNECT): nodes forwarding results of the event.

Local decision node has three decision-making results (RESULT): decision scope is in the area of the event, decision scope is on the boundary of the event area, and no events are in the decision scope.

Initially, all nodes are SLEEP, and the network performs CETC algorithm, execution time is *T*_*c*_, when the algorithm ends, nodes start to detect the event in the monitoring area, and the time of each round is the *T*_*R*_.

(*1) Selecting V*_*D*_. First of all, determine which nodes can be* k-watching* decision nodes in the network. Assume that node *u* integrated a variety of sensors *C*_1_, *C*_2_,…, *C*_*l*_, so *u* with *R*_*si*_/*η*  (1 ≤ *i* ≤ *l*) for the radius broadcast decision node to discover data packets (LDN packet), which includes the ID of the node, the type of sensor *C*_*i*_ integrated by the node, and the residual energy of the node. After time *T*_*D*_, each node receives the LDN data packet from the neighboring nodes, extracts the information of LDN packets, and stores it. Each node will determine whether received at least *k* LDN packets sent by nodes integrated of *C*_*i*_  (1 ≤ *i* ≤ *m*); if so, then the node will be the* k*-watching decision node; the set which consists of* k*-watching decision nodes is denoted as *V*_*CBD*_.

Then, selecting a part of nodes from *V*_*CBD*_ to constitute *V*_*D*_ so that *V*_*D*_ is a dominating set of the graph *G*_*D*_, nodes of *V*_*D*_ will be the local decision nodes. Local decision node has more residual energy compared to other nodes, and there should be more nodes within its decision scope, namely, greater degree. The degree of the node means the number of neighbor nodes; the degree of the node *u* in figure *G* is |*N*_*G*_(*u*)|. Choosing the node with greater degree contributes to reducing the number of local decision nodes in network and making the distribution of local decision nodes more dispersed. The priority Prio represents the residual energy of the node and the whole situation of the degree; the calculation of the priority of *u* is given by

(7) $$\begin{array}{c} {\text{Prio}}_{u}=\frac{E_{\text{residual}}}{E_{\max}}\times \frac{d_{u}}{\overset{-}{d}}, \end{array}$$

where *E*_residual_ denotes the remaining energy of the node, *E*_max⁡_ is the initial energy of the node, *d*_*u*_ is the degree of the node, and $\overset{-}{d}$ indicates the average degree of the nodes in graph *G*_*D*_. Consider

(8) $$\begin{array}{c} \overset{-}{d}=\left\lfloor \left(N-1\right)\times \frac{\pi R_{d}^{2}}{A}\right\rfloor , \end{array}$$

where *N* is the number of nodes in a network and *A* is the size of the monitoring area. Node broadcasts neighbor discovery packets (LN packets) with radius *R*_*d*_ after confirming whether the node itself is* k*-watching decision node; the message contains the ID of the node and whether it is* k*-watching node. After time *T*_*N*_, all* k*-watching decision nodes received LN packets sent from nodes within the scope of *R*_*d*_. In this way, each of the* k*-watching decision nodes will know its own degrees; then the node counts their priority Prio.

*k*-watching decision nodes broadcast the priority packet (PP packet) with *R*_*d*_ radius; the priority information is contained in PP data packet. After *T*_*P*_ time, each node receives packets sent by the* k*-watching decision nodes in the range of *R*_*d*_.* k*-watching decision nodes extract the information of all nodes in PP data packets and then calculate the waiting time of sending BDN packets. The waiting time of a* k*-watching decision node *u* before sending BDN packets is *t*_*u*_:

(9) tu={(1−⌊((Priou−Priomin⁡)/(Priomax⁡−Priomin⁡))×L⌋L) ×  Tτ+Trandom,U  received  PP  packetsTrandom,U  did  not  receive  PP  packets,

where Prio_max⁡_ is the maximum priority among the PP packet received by *u* and Prio_min⁡_ is the minimum priority among the PP packet received by *u*. *T*_*τ*_ is a given time period, *T*_*τ*_ is divided into *L* segments, and *T*_random_ is a random moment in [0, *Tτ*/*L*]. If *u* received a PP packet, the wait time is calculated by the first equation; if *u* does not receive a PP packet, *u* will choose a random value in [0, *Tτ*/*L*] as the waiting time. After time *Ts*, all nodes have finished the calculation and start timing. Waiting for time *t*_*u*_, *u* will send BDN packets. During the waiting period, if *u* received the BDN data packet from other nodes, it will give up sending operation. Otherwise, the node sends a BDN packet and informs that it will become the local decision node and set its STATE to DECIDE.

If a non-*k*-watching decision node *v* does not accept BDN data packets sent from other nodes in time (1 + 1/*L*)*T*_*τ*_, but received PP data packet before, it will send passive local decision node data packets (CDN data packet) to node *w* of maximum priority in PP data packets and inform *w* to be the local decision node; *w* will set the STATE to DECIDE after receiving the message. If being not received PP packet, then *v* is not in the scope of decision node of* k*-watching decisions nodes; thereby there is a detect void in position *v*.

(*2) Selecting V*_*S*_. As local decision nodes have integrated limited sensors and need to select the surrounding nodes as the sensing nodes to provide the environmental information that could not be detected, there is a sensor node list in the cache of the local decision node, storing related information of the sensor node and containing ID of the node and property *A*_*i*_. When *P*(*x*_*i*_) = *true*, the sensing nodes will send simple events data packet (SE packet) to the local decision node in the list.

When choosing *V*_*D*_, each local decision node has received the LDN data packet sent from the neighboring nodes. Local decision node *v* extracts node information from the received the LDN data packet and merges the same information. If node *u* can provide information *A*_1_ and information *A*_3_, then combine the two kinds of information to the perception information of node *u*. Each kind of sensor *C*_*i*_  (1 ≤ *i* ≤ *m*) has a counter count_*i*_; initially, count_*i*_ = *k*. The value of the corresponding count_*i*_  (*j* ≤ *i* ≤ *l*) of the integrated sensor will be minus 1 by *v*; then *v* will sort node information in the LDN data packet by residual energy of the node from large to small. Selecting nodes from the sorted sequence sequentially, let the node selected currently be *u*; if the corresponding count_*i*_ of the integrated sensor of *u* is larger than 0, then *v* will choose *u* as the sensing node to provide the information of attribute *A*_*i*_, and the corresponding count_*i*_  is minus 1. If the corresponding count_*i*_ of all sensors integrated by *u* is 0, then *v* will not select *u* as the sensor node. All of the count_*i*_ have become 0, *v* will cease to choose. When node *v* has selected the sensor node, *v* will send data packets (SN packet) to the selected sensing node; SN data package includes node ID and the list *A*_1_, *A*_2_,…, *A*_*l*_ of attributes to be detected. The sensor node extracts the information from the received SN data packet, adds the source node sending a SN packet to its local decision node list, and then returns the acknowledgment packet (CF packet). Local decision node will add the information of the sensor node to its local decision node list after receiving a confirmation message. Let *T*_*SS*_ be the time required for selecting sensor nodes. The pseudocode of CETC algorithm is as shown in Algorithm 1.

In the initial conditions, the RESULT of the local decision node is Out_Event. If there is part of *e*_*i*_ = 1, but *E* = 0, it indicates the decision range of the local decision node on the boundaries of the Composite event and sets the RESULT of the local decision node as Boundary_Event. If *E* = 1, set the RESULT of local decision node as In_Event. If the RESULT is not Out_Event, the local decision node will send event data packets (CE) to the base station; the data packets contain the position and the RESULT of the local decision node. The base station will determine the area of Composite event according to the positions and decision contained in the received CE data packet.

## 6. Cluster Analysis and Fault Detection

Supposing the transmit period of perception nodes is *T*_*s*_, the local decision node receives the data transmitted by the perception node periodically. It needs to detect *m* kinds of attributes of the environment in order to determine the Composite events. The decision ranges of local decision making nodes satisfy* k*-watching nature; that is to say, the local decision node will receive *m* × *k* data each cycle. We use the sliding window mechanism, making the local decision node stores the data of the recent *p* cycles at any moment. That means the size of the sliding window is *p*, so it saves *p* × *m* × *k* data each moment. The value of *j*th attribute at time *i*  (1 ≤ *i* ≤ *p*) of the sliding window is *A*_*j*_^*i*^ = {*x*_*j*1_, *x*_*j*2_,…, *x*_*jk*_}, the data received at time *i*  (1 ≤ *i* ≤ *p*) is *A* ^*i*^ = {*A*_1_^*i*^, *A*_2_^*i*^,…, *A*_*m*_^*i*^}, and the data of the *j*th attribute of the local decision node is *A*_*j*_ = {*A*_*j*_^1^, *A*_*j*_^2^,…, *A*_*j*_^*p*^}. Data received is *A* = {*A* ^1^, *A* ^2^,…, *A* ^*p*^} and carrying out cluster analysis on *A*_*j*_^*i*^ and *A*_*j*_, algorithm including the two clustering analysis is called two-dimensional *τ*-GAS algorithm. GAS (generalized agglomerative scheme) [34] is a kind of hierarchical clustering mechanism; two-dimensional *τ*-GAS algorithm adds restriction conditions on the basic of the GAS algorithm and uses spatiotemporal correlation to cluster the data.

Initially, each attribute value corresponds to a class, a total of *k* classes, as *R*_0_ = {*C*_*l*_ = {*x*_*l*_}, *l* = 1,2,…, *k*}; the classification obtained at step *q* is denoted as *R*_*q*_. Then, the clustering of the first layer data is carried out. Due to spatiotemporal correlation, we mark the differences between the two classes using the poor between the two elements with the smallest difference in two classes, denoted as *g*(*C*_*n*_, *C*_*q*_):

(10) $$\begin{array}{c} g\left(C_{n},C_{q}\right)=\min\left(\left|x_{i}-x_{j}\right|\right),\;\left(x_{i}\in C_{n},\;\;x_{j}\in C_{q}\right). \end{array}$$

Then merge the smallest two classes of *g*(*C*_*n*_, *C*_*q*_) and get *C*_*r*_ = *C*_*n*_ ∪ *C*_*q*_. Using span *ψ* to denote the maximum difference between the elements in a class as follows:

(11) $$\begin{array}{c} \psi_{C_{r}}=\max\left(\left|x_{i}-x_{j}\right|\right),\;\left(x_{i},x_{j}\in C_{r}\right). \end{array}$$

Values of the same attribute are similar; therefore, for the *j* attribute, given a threshold *τ*_*j*_, and *ψ*_*C*_*r*__ ≤ *τ*_*j*_, *τ*_*j*_ could not be too small, in case that reasonable data are neglected, *τ*_*j*_ could not be too large, in case that wrong data and correct data are in a class. When *ψ*_*C*_*r*__ ≤ *τ*_*j*_, merge two minimum classes of *g*(*C*_*n*_, *C*_*q*_) and get *R*_1_ = (*R*_0_ − {*C*_*n*_, *C*_*q*_}) ⋃ {*C*_*r*_}. Then repeat the above steps until merging of any two of the classes does not satisfy the formula *ψ*_*C*_*r*__ ≤ *τ*_*j*_, or leaving only one classify finally.

Setting the class *C*_*ε*_ with maximum number of elements as a correct data class, the average ${\overset{-}{x}}_{j}^{\;i}$ is the value of attribute *j* at time *i*:

(12) $$\begin{array}{c} {\overset{-}{x}}_{j}^{\;i}=\frac{1}{\left|C_{e}\right|}\overset{}{\underset{x_{i}\in C_{e}}{\sum }}x_{i}. \end{array}$$

If the number of category *C*_*ε*_ with maximum number of elements is more than one, then calculate ${\overset{-}{x}}_{j}^{\;i}$ of each classification; finally, take values which are most close to event attribute, and the corresponding class will be attributed to the correct category. As classifying temperature data in fire detection, if the number of category *C*_*ε*_ with maximum number of elements is more than one, then the class *C*_*ε*_ with the largest ${\overset{-}{x}}_{j}^{\;i}$ is considered as a correct category. The average of all the *j*th properties at *p* moments is ${\overset{-}{A}}_{j}=\{{\overset{-}{x}}_{j}^{\;1},{\overset{-}{x}}_{j}^{\;2},\ldots ,{\overset{-}{x}}_{j}^{\;p}\}$. In order to eliminate the temporary failure of the node, cluster the data of ${\overset{-}{A}}_{j}$ by the process above and get the average ${\overset{-}{x}}_{j}$. The algorithm above is called two-dimensional *τ*-GAS algorithm; the description of the algorithm is as shown in Algorithm 2.

Next, we present an example of performing a two-dimensional *τ*-GAS cluster on temperature.  Table 1 shows a set of temperature data when *p* = 5, *k* = 6.

Analyzing the group of data with two-dimensional *τ*-GAS algorithm. According to the algorithm, it will analyze the data of every moment. Next we will process the data at the third moment:

(13) At3={55.7,81.2,54.1,55.6,21.6,55.1},  τt=10°C.At3{{x1},{x2},{x3},{x4},{x5},{x6}}R0{{x5},{x3},{x6},{x4},{x1},{x2}}R1{{x5},{x3},{x6},{x4,x1},{x2}}R2{{x5},{x3},{x6,x4,x1},{x2}}R3{{x5},{x3,x6,x4,x1},{x2}}.

**Figure 2:**
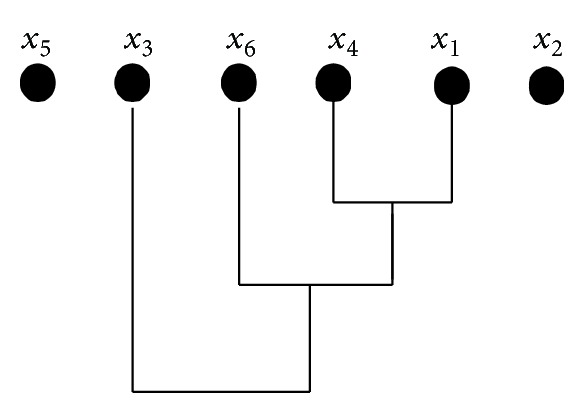
Clustering process.

Figure 2 is the clustering process of *A*_*t*_^3^ with three steps in total; we get *R*_0_, *R*_1_, *R*_2_, and *R*_3_ after clustering. The number of elements in {*x*_2_, *x*_6_, *x*_4_, *x*_1_} of *R*_3_ is the largest; therefore, *x*_2_ and *x*_5_ are considered to be unreliable; ${\overset{-}{x}}_{t}^{3}=55.13$. Analyzing the other data, we get ${\overset{-}{A}}_{t}=\{54.98,55.02,54.98,55.02,54.53\}$. Then clustering ${\overset{-}{A}}_{t}$ again, we will get the class {54.53,54.98,55.02,55.13} with the most elements, 29.52 may be caused by the temporary changes of the environment or temporary failure of the node. Therefore, ${\overset{-}{x}}_{t}=54.92$ is used to indicate the current temperature of the environment.

The *τ*-GAS function will sort the collected data first; the complexity of the algorithm is *O*(*k*log⁡⁡*k*). In the layer *t*, it is needed to compare for *k* − *t* − 1 times; the comparison number of the algorithm is

(14) $$\begin{array}{c} \overset{k-1}{\underset{t=0}{\sum }}\left(k-t-1\right)=\frac{k\left(k-1\right)}{2}. \end{array}$$

Then, the total time complexity of the two-dimensional *τ*-GAS algorithm is *O*(*p* × *k* ^2^ + *p* ^2^).

As for *A*_*j*_^*i*^ = {*x*_*j*1_, *x*_*j*2_,…, *x*_*jk*_}, if *C*_*e*_ = {*x*_*jq*_, *x*_*j*2_,…, *x*_*jl*_}, then the node producing the data {*x*_*j*1_, *x*_*j*2_,…, *x*_*j*(*q*−1)_,…, *x*_*j*(*l*+1)_,…, *x*_*jk*_} is considered to fail. The node will be informed by the local decision node. Before running CETC algorithm, node sets *F*_*i*_ = 0 for each sensor, *F*_*i*_ represents the times of failure of the node in a round. If local decision node determines that a sensory data is invalid, then it will notify the corresponding nodes, value *F*_*i*_ of the corresponding node increases 1. Time of each round is *T*_*R*_, the execution time of CETC algorithm is *T*_*c*_, and data collection cycle of local decision node is denoted by *T*_*E*_; then the collecting times of each round are

(15) $$\begin{array}{c} N=\frac{T_{R}-T_{C}}{T_{E}}. \end{array}$$

The perception probability of a sensor node failure is

(16) $$\begin{array}{c} p_{f}=\frac{F_{i}}{N}=\frac{F_{i}\times T_{E}}{T_{R}-T_{C}}. \end{array}$$

Given a threshold *θ*, if *p*_*f*_ > *θ*, the node cannot sense the environment.

## 7. Fuzzy Decision of Composite Events

Fuzzy decision is an important part of fuzzy system theory. A decision is the countermeasures and strategies taken on a thing [35]. In real life, factors that affect the determine results of Composite events are fuzzy mostly, a given method is sorted by fuzzy decision algorithm; decision results can be considered as solution set, from which the most appropriate processing scheme is selected. Such as in fire detection, the decision results of the node are divided into three possible schemes {send information of event occurs, send information of the abnormal environment, without any operation}. In some cases, part of the property value of the environment is higher, but the event has not occurred; then the scheme of abnormal environment was sent. For this kind of abnormal phenomenon, we will process it additionally.

The estimate value $\left\{{\overset{-}{x}}_{1},{\overset{-}{x}}_{2},\ldots ,{\overset{-}{x}}_{m}\right\}$ of the current attribute of the environment can be obtained by the two-dimensional *τ*-GAS algorithm; with the estimated value, we can obtain the optimal scheme of the event decision. In the paper, we adopt a compromised type of fuzzy multiattribute method [36] to make decisions on events; its basic idea is that for the initial sample data, virtual fuzzy positive ideal solution, and fuzzy negative ideal solution, the fuzzy positive ideal solution is constituted of the maximum value of the fuzzy parameter values of each attribute, and the fuzzy negative ideal solution is constituted of the minimal value of fuzzy indicator values. Then calculate the weighted distance between each alternative and the fuzzy positive ideal solution and fuzzy negative ideal solution with weighting method. On this basis, calculate the membership degree of each alternative belonging to fuzzy positive ideal; the greater the degree of membership, the more ideal the scheme.

The paper modifies the compromised type of fuzzy multiattribute method appropriately to adapt the determination of the Composite event in the wireless sensor network. The set of the attributes of the determining solution is *V* = {*V*_1_, *V*_2_,…, *V*_*m*_}, the set of node decision scheme is *A* = {*A*_1_, *A*_2_,…, *A*_*n*_}, elements of *A* are ordered, *V*_*i*_ is the factor that the scheme needs to consider, such as fire detection, and the *V*_*i*_ can be temperature; it can also be a temperature of fixed period of time. *A*_*i*_ indicates node processing solution: choosing the optimal solution by decision algorithm. The following is the main steps of the determining mechanism based on the fuzzy decision.

**Step 1 .**

The membership degree function matrix of each attribute relative to the scheme is *I* = (*u*_*ij*_(*x*))_*n*×*m*_, and calculate the fuzzy indicators matrix *F* = (*f*_*ij*_)_*n*×*m*_:

(17) $$\begin{array}{c} I=\left(\begin{array}{cccc} u_{11}\left(x\right) & u_{12}\left(x\right) & \cdots & u_{1m}\left(x\right)\\ u_{21}\left(x\right) & \cdots & \cdots & u_{2m}\left(x\right)\\ \vdots & \vdots & \vdots & \vdots \\ u_{n1}\left(x\right) & u_{n2}\left(x\right) & \cdots & u_{nm}\left(x\right) \end{array}\right). \end{array}$$

*u*_*ij*_(*x*) is a membership function, indicating the suitability of adopting scenario *i* when the value of *j*th attribute is *x*. Membership function can be formulated by experts of the relevant application domain or obtained by machine learning algorithms. Given $\left\{{\overset{-}{x}}_{1},{\overset{-}{x}}_{2},\ldots ,{\overset{-}{x}}_{m}\right\}$, calculate the fuzzy indicators matrix *F*:

(18) $$\begin{array}{c} F=\left(\begin{array}{cccc} u_{11}\left({\overset{-}{x}}_{1}\right) & u_{12}\left({\overset{-}{x}}_{2}\right) & \cdots & u_{1m}\left({\overset{-}{x}}_{m}\right)\\ u_{21}\left({\overset{-}{x}}_{1}\right) & \cdots & \cdots & u_{2m}\left({\overset{-}{x}}_{m}\right)\\ \vdots & \vdots & \vdots & \vdots \\ u_{n1}\left({\overset{-}{x}}_{1}\right) & u_{n2}\left({\overset{-}{x}}_{2}\right) & \cdots & u_{nm}\left({\overset{-}{x}}_{m}\right) \end{array}\right). \end{array}$$

**Step 2 .**

Weigh vector *w* = {*w*_1_, *w*_2_,…, *w*_*m*_}; the weight vector *w* reflects the influence of different attributes on scheme selection.

**Step 3 .**

Construct fuzzy decision matrix *D* = (*r*_*ij*_)_*n*×*m*_, where *r*_*ij*_ = *w*_*j*_ × *f*_*ij*_.

**Step 4 .**

Define the fuzzy positive ideal solution *M* ^+^ and fuzzy negative ideal solution *M* ^−^, where *M* ^+^ = (*M*_1_^+^, *M*_2_^+^,…, *M*_*m*_^+^) and *M* ^−^ = (*M*_1_^−^, *M*_2_^−^,…, *M*_*m*_^−^). Among this, *M*_*j*_^+^ = max⁡{*r*_1*j*_, *r*_2*j*_,…, *r*_*nj*_}, *j* = 1,2,…, *m* is the maximum value relative to indicator values of the *j*th column. The fuzzy index *M*_*j*_^−^ = min⁡{*r*_1*j*_, *r*_2*j*_, , *r*_*nj*_}  (*j* = 1,2,…, *m*) is the minimal value relative to fuzzy indicator values of the *j*th column.

**Step 5 .**

Calculate the distance *d*_*i*_^+^ between scheme *A*_*i*_ and fuzzy positive ideal solution *M* ^+^ and the distance *d*_*i*_^−^ between scheme *A*_*i*_ and fuzzy negative ideal solution *M* ^−^, respectively:

(19) di+=∑j=1m(rij−Mj+)2, i=1,2,…,ndi−=∑j=1m(rij−Mj−)2, i=1,2,…,n.

**Step 6 .**

Select decision scheme.

Let a scheme *A*_*i*_ belong to the fuzzy positive ideal with membership *u*_*i*_; here

(20) $$\begin{array}{c} u_{i}=\frac{d_{i}^{-}}{d_{i}^{+}+d_{i}^{-}}. \end{array}$$

Clearly, 0 ≤ *u*_*i*_ ≤ 1, if the *A*_*i*_ is more close to *M* ^+^, the *u*_*i*_ is more close to 1. According to the membership degree, sort *u*_*i*_ in descending order. The greater the *u*_*i*_ is, the more reasonable the scheme *A*_*i*_ is. Scheme of *A* is sorted from high to low order according to the urgency; when *u*_*i*_ = *u*_*j*_ = max⁡{*u*}  (*i* ≠ *j*), if *i* < *j*, select *A*_*i*_; otherwise, select *A*_*j*_.

There are two important parameters in the algorithms above: the set of membership functions *I* = (*u*_*ij*_(*x*))_*n*×*m*_ and weight vector *w*. The algorithm can be suitable for the Composite event detection in different environments by configuring different *I* and *w*. Such as fire detection, the environment in summer and winter is different. The temperature in summer is higher; modifying the membership functions associated with temperature and cut down the weight of temperature can reduce the effect of temperature on scheme selection. The temperature in winter is lower; raising the weight of temperature can improve the effect of temperature on scheme selection.

The decision mechanism based on threshold is unable to process the fuzzy properties. The rule base grows exponentially as the attribute increases in the decision mechanism based on fuzzy logics. The proposed decision mechanism of Composite event based on fuzzy logic can not only deal with the vaguely defined attribute but also with the condition that the number of elements in the membership function matrix *I* grows exponentially as *m* increases when *n* is given.

For scheme *A*_*i*_, if part of *r*_*ij*_ is bigger, then *u*_*i*_ will be larger. The determination mechanism based on fuzzy decision cannot indicate the complex Boolean function in the determination mechanism based on threshold and complex rules in the decision mechanism based on fuzzy logic; thus, it cannot judge the more complex Composite event. When most attributes of the environment or part of properties with greater weight meet the requirements of the occurrence of Composite event, the determination mechanism based on fuzzy decision can determine whether the Composite event occurs, which meets the nature of most Composite events in the real world; the rules package in the literature [21] contains such rules. Therefore, the determination mechanism based on fuzzy decision of the Composite event can be applied to the Composite event detection in the general situation.

## 8. Performance Analysis

This section will discuss the time complexity and the complexity of message exchange of the algorithm and analyze advantages of detecting events using multiple kinds of properties. Finally, it will discuss the selection of *η*.

### 8.1. Complexity and Performance Analysis of the Algorithm

**Theorem 2 .**

The CETC algorithm would end within a limited time.

**Proof**

**Figure 3:**
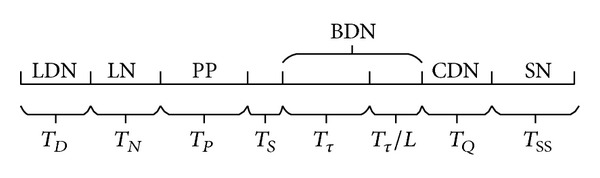
Time periods of CETC algorithm execution.

From CETC algorithm, we can know that the algorithm is time synchronization. The algorithm has eight time periods corresponding to different calculation and operations of data sending. As shown in Figure 3, scale above marks data packets to be sent, and scale below is the corresponding time period. If exceeding the corresponding time period, the node will not deal with the corresponding data packets. Therefore, CETC algorithm will end within *T*_*D*_ + *T*_*N*_ + *T*_*P*_ + *T*_*S*_ + *T*_*τ*_ + *T*_*τ*_/*L* + *T*_*Q*_ + *T*_*SS*_.

**Theorem 3 .**

The total time complexity of CECT algorithm is *O*(Δ′ + Δlog⁡⁡(Δ)), where Δ indicates the maximum value of the number of nodes in the scope 1/*η*max⁡{*R*_*si*_}  (*i* = 1,2,…, *m*) of network node and Δ′ shows the maximum number of nodes in the decision range of any node in the network, that is, the maximum node degree in figure *G*_*D*_.

**Proof**

In Figure 3, the corresponding calculation operations must be carried out at each time period. In the time period *T*_*D*_, the time complexity of broadcasting LDN packet of each node is *O*(1). In the time period *T*_*N*_, node needs to decide whether it can be a* k-watching* decision node; therefore, nodes need to deal with the received LDN packets one by one in the time period *T*_*D*_ and the time complexity is *O*(Δ). In the time period *T*_*p*_, nodes need to calculate their own priorities and the time complexity is *O*(1). In the time period *T*_*S*_, nodes need to calculate the time of sending BDN data packet, so they need to find the maximum value and minimum value of priority level of the received PP data packet, and the time complexity is *O*(Δ′). BDN data packets will be sent and received in the time period *T*_*τ*_ + *T*_*τ*_/*L*; the time complexity is *O*(1). In the time period *T*_*Q*_, non-*k*-watching decision node that did not receive BDN data packet needs to look for the maximum priority level of the received PP data packet; the time complexity is *O*(Δ′). In the time period *Ts* *s*, local decision node needs to extract the information of the node from LDN data packet and sorts nodes according to the energy of the node; then choosing the node with larger energy as sensor node disposable, the time complexity of sorting is *O*(Δlog⁡⁡(Δ)). So CETC algorithm's total time complexity is *O*(Δ′ + Δlog⁡⁡(Δ)).

**Theorem 4 .**

Message exchange complexity (Message exchange complexity) of CETC algorithm is *O*(*N*) and *N* is the total number of nodes in a network.

**Proof**

In addition to exchanging message with specific node in the *T*_*Q*_ and *T*_*SS*_ period, nodes carry out broadcasting operations in the other period, so message exchange complexity of each node is *O*(1). So, the message complexity of the entire network is *O*(*N*).

**Theorem 5 .**

If nodes in the network are all* k*-watching decision nodes, when the CETC algorithm ends, the probability of two local decision nodes' distance less than *R*_*d*_ is small.

**Proof**

If nodes in the network are all* k*-watching decision nodes, this means that each node can send BDN package. In the time period *T*_*τ*_ + *T*_*τ*_/*L*, all the nodes could be controlled by the local decision node. Assume that there are two nodes *u* and *v*, whose distance is not larger than *R*_*d*_, the waiting time before *u* sends BDN data packet is as follows:

(21) tu=(1−⌊((Priou−Priomin⁡)/(Priomax⁡−Priomin⁡))×L⌋L) ×Tτ+Trandom.

As seen from the above equation, *T*_*τ*_ is divided into *L* segments; each node selects a time period according to the priority firstly and then chooses a random time *T*_random_ within this time period, *T*_random_ ∈ [0, *T*_*τ*_/*L*]. Because of the uncertainty in figure *G*_*D*_ and residual energy of *u* and *v*, and the priority of Prio is uncertain, *u* and *v* will choose a moment randomly in time period *T*_*τ*_ + *T*_*τ*_/*L*. Assume that there is *K* moments within *T*_*τ*_/*L*. So the probability of node sending BDN data packets at any moment is as follows:

(22) $$\begin{array}{c} P_{u}=P_{v}=\frac{1}{K\left(L+1\right)}. \end{array}$$

If *u* and *v* are local decision nodes, then *u* and *v* will send BDN data packets at the same time; the probability is

(23) $$\begin{array}{c} P=P_{u}\times P_{v}=\frac{1}{K^{2}{\left(L+1\right)}^{2}}. \end{array}$$

When *K* = 10 and *L* = 10, *P* ≈ 8.3 × 10^−5^. In fact, the value of *K* is bigger and the usage of collision detection algorithm would also be considered; as a result, the probability of the distance of two local decision nodes less than *R*_*d*_ is small.

Theorem 5 shows that local decision node is geographically distributed when CECT algorithm ends; thus, it helps to balance energy consumption of the network.

**Theorem 6 .**

When voids are not detected, *V*_*D*_ is a dominating set of graph *G* = (*V*, *E*).

**Proof**

The CETC algorithm shows that if the node sends BDN packets successfully, then STATE of the node is set to DECIDE. If *u* does not send or receive any BDN data packets, then *u* is not available* k-watching* decision node; the node will look for node *v* with biggest Prio within the scope of *R*_*d*_ as a local decision node. When voids are not detected, *u* always can find such a node *v*. So for any node in the network, a local decision node always can be found within the scope of *R*_*d*_. It can be seen that *V*_*D*_ is a dominating set of graph *G*_*D*_ = (*V*, *E*_*D*_). Due to *E*_*D*_⊆*E*, *V*_*D*_ is a dominating set of graph *G* = (*V*, *E*).

From Theorem 6, we know that when the voids are not detected, the decision range of local decision node covers all nodes within the monitoring area, so nodes in the *V*_*D*_ + *V*_*S*_ are active; then they can monitor all Composite events in the position of the nodes, so as to monitor the occurrence situation of Composite event in the whole monitoring area approximately.

**Theorem 7 .**

The set constituted of all active nodes is connected dominating set of *G*′ = (*V*′, *E*′).

**Proof**

The DS algorithm shows that the set constituted of all active nodes is *V*_*D*_ ∪ *V*_*S*_ ∪ *V*_*C*_ ∪ {sin*k*}. In the network, any local decision node *u* sends activation message to its parent nodes *u*_*p*1_, *u*_*p*2_, and the activation message will be transmitted to the base station. Nodes on the Path *u* → sin*k* could only be local decision nodes, sensing nodes, or connection nodes; connection nodes are only possible on such path.

Accordingly, in the case that graph *G*′ = (*V*′, *E*′) is connected, there is at least one path between local decision nodes and base station. Since *V*_*D*_ is the dominating set of graph *G* = (*V*, *E*), so the *V*_*D*_ ∪ {sin*k*} is the dominating set of graph *G*′ = (*V*′, *E*′). Because *V*_*S*_ and *V*_*C*_ ensures the connectivity from nodes in *V*_*D*_ to the base station, so all the active nodes *V*_*D*_ ∪ *V*_*S*_ ∪ *V*_*C*_ ∪ {sin*k*} are the connected dominating set of graph *G*′ = (*V*′, *E*′).

Theorem 7 shows that the connection node ensures the connectivity between local decision nodes and base stations, so the information of Composite event of the local decision node can be transmitted to the base station; if nodes in *V*_*D*_ ∪ *V*_*S*_ ∪ *V*_*C*_ are active, then they can complete the detection of events within the monitoring area and send the result of the local decision to the base station.

### 8.2. Analysis of Composite Events

**Theorem 8 .**

Testing events with multiple attributes are more accurate than with one attribute.

**Proof**

Assuming that Composite event is *E*, if using *M* properties can completely test *E*. Now there are only *m* ≤ *M* kinds of sensors in the network; the judgment of the *i*th property is *e*_*i*_. *e*_*i*_  (1 ≤ *i* ≤ *m*) are not related; then {*E* = 1} = {*e*_1_ = 1}∩{*e*_2_ = 1}∩⋯∩{*e*_*M*_ = 1}.

Because {*e*_1_ = 1}∩{*e*_2_ = 1}∩⋯∩{*e*_*M*_ = 1}⊆{*e*_1_ = 1}∩{*e*_2_ = 1}∩⋯∩{*e*_*m*_ = 1}, then

(24) $$\begin{array}{c} \left\{E=1\right\}\subseteq \left\{e_{1}=1\right\}\cap \left\{e_{2}=1\right\}\cap \cdots \cap \left\{e_{m}=1\right\}. \end{array}$$

When *e*_*i*_ = 1, the occurrence probability of composite event probability of {*E* = 1} is as follows:

(25) P({E=1} ∣ {ei=1})=P({E=1}∩{ei=1})P({ei=1})=P({E=1})P({ei=1}).

When testing various attributes, when *e*_1_ = 1, *e*_2_ = 1,…, *e*_*m*_ = 1, the probability of the Composite event is as follows:

(26) P({E=1} ∣ {e1=1}∩{e2=1}∩⋯∩{em=1}) =P({E=1}∩{e1=1}∩{e2=1}∩⋯∩{em=1})P({e1=1}∩{e2=1}∩⋯∩{em=1}) =P({E=1})∏i=1mP({ei=1}).

In order to simplify the analysis, *P*({*e*_*i*_ = 1}) = *p*_*e*_  (1 ≤ *i* ≤ *M*); then

(27) κ=P({E=1} ∣ {e1=1}∩{e2=1}∩⋯∩{em=1})P({E=1} ∣ {ei=1})=1pem−1=(1pe)m−1.

If *P*_*e*_ is identified, 1/*P*_*e*_ > 1, *k* grows exponentially along with *m*. Therefore, testing the event with multiple attributes is more accurate than with one attribute.

In the ideal state, no event occurs, *e*_*i*_ = 0; when the event occurs, *e*_*i*_ = 1. In real environment, the situation that {*e*_*i*_ = 1∣*H*_0_} and {*e*_*i*_ = 1∣*H*_1_} tends to appear, which is called that the node has been influenced. There may be two situations of the interference: the first is the failure of the node itself; the second is the interference caused by other factors in the environment. For example, in forest fire detection, temperature properties are *T*; when {*T* = 1∣No  Fire}, a node may fail, or the sensor is exposed to direct sunlight in summer, causing the perceived temperature data to be higher. *E* is the real value whether events happen in the environment; *R* is the determination result that according to *m* types of properties values of the event of the node, {*R* = 1} = {*e*_1_ = 1}∩{*e*_2_ = 1}∩⋯∩{*e*_*m*_ = 1}.

Setting the probability that the node is interfered as *p*, *p* = *P*({*e*_*i*_ = 1}∣*H*_0_) = *P*({*e*_*i*_ = 0}∣*H*_1_), and the probability of the nodes correctly detecting the environment is as follows:

(28) pd=P({R=1} ∣ H1)×P(H1)+P({R=0} ∣ H0)×P(H0)=(1−p)mpE+(1−pm)(1−pE).

The probability that node cannot detect the environment correctly is as follows:

(29) pf=P({R=1} ∣ H0)×P(H0)+P({R=0} ∣ H1)×P(H1)=(1−(1−p)m)pE+pm(1−pE).

The *P*_*E*_ indicates the probability that event occurs. For given *p* and *P*_*E*_, the influence that *m* has on *P*_*d*_ is analysed. Making derivation for *m*, when the derivative is zero, get *m* when *P*_*d*_ is the maximum value:

(30) $$\begin{array}{c} m=\frac{\ln\left(\left(\left(1-p_{E}\right)\ln p\right)/\left(p_{E}\ln\left(1-p\right)\right)\right)}{\ln\left(1-p\right)-\ln\left(p\right)}. \end{array}$$

**Figure 4:**
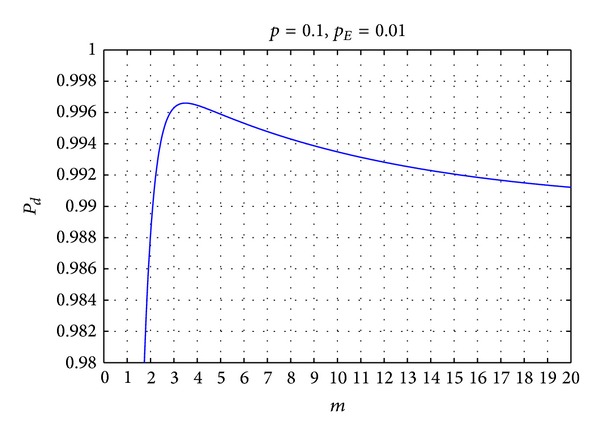
The relationship between *P*_*d*_ with *m*.

*P*_*d*_ first increases and then decreases along with *m*.  Figure 4 is the situation of *P*_*d*_ when *p* = 0.1, *P*_*E*_ = 0.

If the detection is required with higher accuracy, *m* can take larger integers around the extreme value *P*_*d*_. But for the important event, it is considered that the node can detect the change of attributes of the environment and has believable probability of determination result when the events occur; that is,

(31) PEd=P({E=1} ∣ {R=1})×P({R=1} ∣ H1)×P(H1)=PEpem×(1−p)mpE=pE2(1−ppe)m.

So if testing important events, when (1 − *p*)/*P*_*e*_ < 1, *m* should be as small as possible, but in order to ensure that *P*_*d*_ is larger, *m* should take larger integers around the extreme value *P*_*d*_. When (1 − *p*)/*P*_*e*_ ≥ 1, *m* is the large the better. When *m* → *∞*, lim⁡_*m*→*∞*_⁡*p*_*d*_ = 1 − *p*_*E*_. Since *P*_*d*_ is monotonically decreasing, so when *m* takes a larger value, the probability of node accurately testing the environment is still great as long as *P*_*E*_ is lesser.

### 8.3. Selection of *η*

According to the CETC algorithm, *R*_*C*_ must meet the conditions that *R*_*C*_ ≥ *R*_*d*_ = (1 − *η*)min⁡{*R*_*Si*_} and *R*_*C*_ ≥ max⁡{*ηR*_*Si*_}  (1 ≤ *i* ≤ *m*); then

(32) $$\begin{array}{c} R_{C}\geq \max\left(\left(1-\eta \right)\min\left\{R_{Si}\right\},\eta \max\left\{R_{Si}\right\}\right)\;\left(1\leq i\leq m\right). \end{array}$$

To simplify the analysis, assume that there are *m* kinds of nodes deployed in area *A* randomly, the *i*th node integrated sensor is  *C*_*i*_ and the number of this kind of nodes is *N*_*i*_. For the node *u* integrated *C*_*i*_, *B*_*i*_ denotes that there is the *i*th node in the range *R* *si*/*η* (*η* is the integer greater than 1) from *u*, and *D* indicates that *u* can be the local decision node; then

(33) $$\begin{array}{c} P\left(B_{i}\right)=1-\overset{N_{i}}{\underset{i=1}{\prod }}\frac{A-\pi \left(R_{Si}/\eta \right)}{A}=1-{\left(\frac{A\eta^{2}-\pi {R_{Si}}^{2}}{A\eta^{2}}\right)}^{N_{i}}. \end{array}$$

(34) $$\begin{array}{c} P\left(D\right)=\left(\overset{m}{\underset{i=1,i\neq l}{\prod }}P{\left(B_{i}\right)}^{k}\right)\times P{\left(B_{l}\right)}^{k-1} \end{array}$$

When deploying nodes, we require that *P*(*D*) ≥ *θ*, where *θ* is the given threshold. For *m*, *N*_*i*_, *R*_*Si*_  (1 ≤ *i* ≤ *m*); *P*(*D*) will decrease along with *n* increasing. When *P*(*D*) = *θ*, we will get a biggest integer *η*, and it is the optimal solution. So *η* makes the decision range biggest and decreases the number of local decision nodes and active nodes. Assuming that *N*_*i*_ = *N*_0_, *R*_*Si*_ = *R*_*S*0_, 1 ≤ *i* ≤ *m*, and *k* = 1, then

(35) P(Bi)=1−∏i=1NiA−π(RS0/η)2A=1−(1−πRS02Aη2)N0P(D)=(1−(1−πRS02Aη2)N0)m−1≥θη=⌊πRS02A(1−(1−θ1/(m−1))1/N0)−1⌋.

## 9. Experiment and Discussion

We do the simulation experiments and analysis about the accuracy, error rate, energy consumption, the fault detection ability, and decision mechanism of the proposed algorithm in Java and MATLAB. We perform a fire detecting in area 300 × 300 m^2^; three kinds of sensor nodes are deployed randomly in the area to perceive the temperature, light intensity, and smoke concentration, respectively, the initial energy of the node is 1 J, and the size of packet is 128 bits. The number of each kind of nodes is *N*_0_; the largest communication radius of the sensor is 40 m, taking *θ* = 90%, *k* = 1. Next we will take simulation experiment under the condition *N*_0_ = 300,400,…, 1300, the corresponding optimal *η* = 2,2, 2,3, 3,3, 3,4, 4,4, 4. The area of Composite event is a circular area with radius 100 m; the Composite event happens randomly. The experimental data is the average of 50 times simulation.

**Figure 5:**
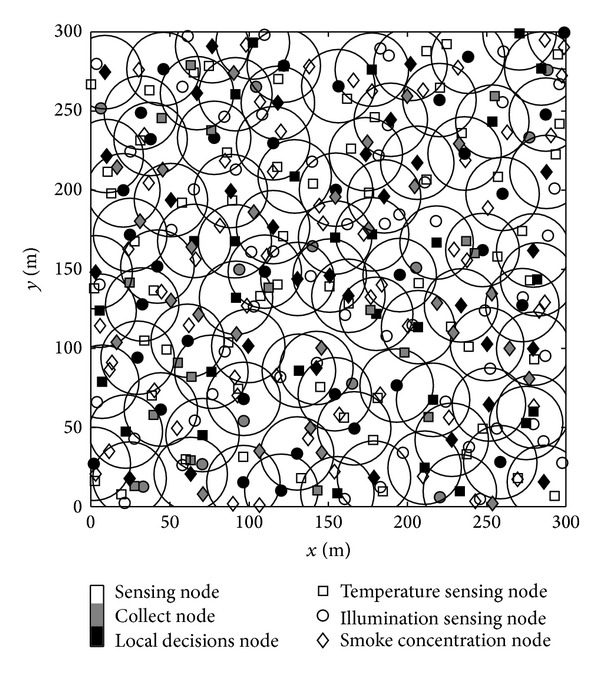
*N* = 300.

Figure 5 is the condition when *N*_0_ = 300, there is a total of 113 local decision nodes, and sleeping nodes are not marked. Distribution of decision node is more scattered. There still exist a few areas that have not been covered, though they almost cover the entire monitoring area. We only need to determinate these nodes to make sure whether there is a fire in the monitoring area. The experimental parameters are shown in Table 2.

### 9.1. Failure Detection

Assume that the probability of node failure is *p*. For the faulty node, it will produce the wrong perception attribute values. We separate the wrong data from the sensing data by the two-dimensional *τ*-GAS algorithm. Next we will analyze the relationship between *K* and detection accuracy and the false alarm rate to shown their influence on the two-dimensional *τ-*GAS algorithm. Precision (DR) is defined as the ratio of the number of the fault nodes detected accurately and the number of actual fault nodes in the network. The false alarm rate (FAR) is the ratio of the number of nonfaulty nodes which are wrongly detected and the number of normal nodes in the network.

**Figure 6:**
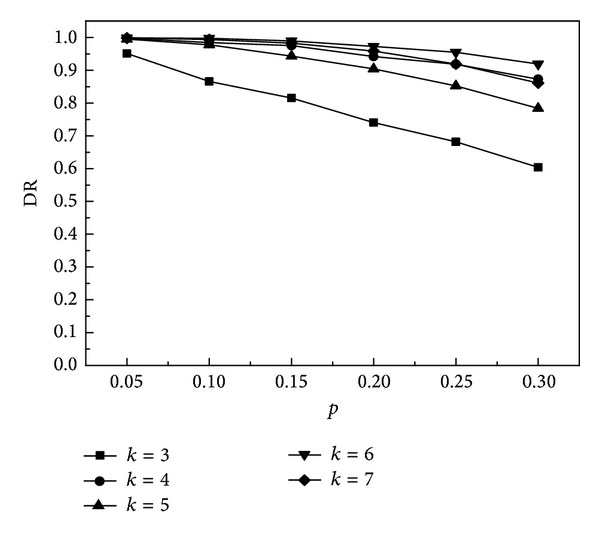
The relationship of DR with *p*.

From Figure 6, when *k* is the same, the larger *p* is and the smaller DR is; that means under the same conditions, detection accuracy will be reduced when there are much fault nodes. When *p* is the same, we found that DR is bigger when *k* is even; this is because such an assumption is made in the simulation that the node can always choose the right category to make fuzzy decision-making when the number of two types of data is the same. From the overall trend, DR increases as *k* increases, and this is because the larger the *k* is, the more the nodes are that will provide the attribute information of the environment; then the detection accuracy will be improved.

**Figure 7:**
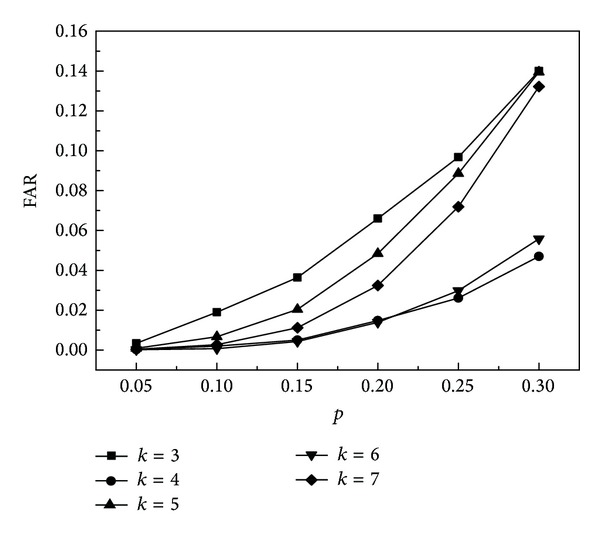
FAR's relationship with *p*.

As can be seen from Figure 7, when *k* is even, the above problem still exists. From the overall trend, FAR decreases along with the increase of *k*. This is because that the greater the *k* is, the more the nodes are that provide the attribute information of the environment, and the error rate will reduce.

By formula (34), when given *η* and the number of nodes in the network, *P*(*D*) monotonically decreases along with the increase of *k*. Since *P*(*D*) ≥ *θ*, the optimal solution of *k* is the max value of *k* when *P*(*D*) ≥ *θ*. In practical applications, the *η* and *k* may all be variable, larger *η* helps to reduce the energy consumption in network; a larger *k* could improve the accuracy of event detection and fault detection. But when given the number of nodes, *P*(*D*) will decrease rapidly along with the increase of *η* and *k*. Therefore, in practice, we should establish a trade-off between energy consumption and detection accuracy and choose reasonable *η* and *k*.

### 9.2. Energy Consumption and Accuracy

This experiment evaluates the energy consumption situation of the detection algorithm through the number of active nodes and the exchange capacity of the information when event occurs. Active node refers to the node which is in a nondormant state; capacity of message exchange refers to the number of all the packets which are received by the nodes in the network and used to detect the event.

**Figure 8:**
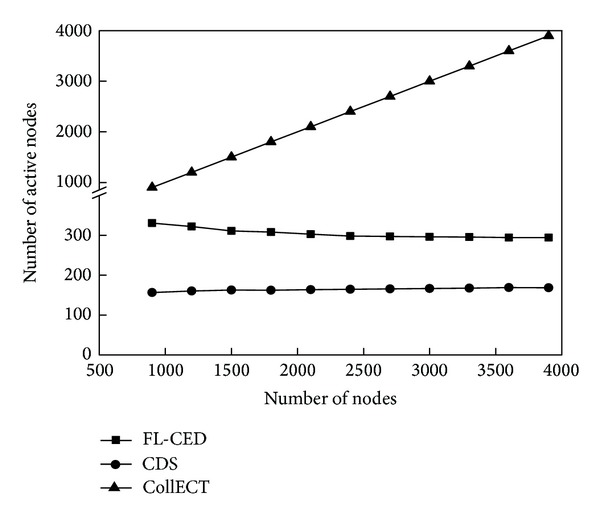
Number of active nodes.

Figure 8 reflects the number of active nodes of the three algorithms. There are more active nodes in CollECT algorithm because of lack of sleep scheduling. FL-CED and CDS algorithms use sleep scheduling mechanism so that the number of active nodes reduced greatly. From the figure, the larger the *η* in FL-CED algorithm is, the smaller the number of active nodes is; this is due to the fact that the greater *η* leads to larger decision range of the node, so the monitoring area could be tested with fewer active nodes in the network. Since the perception range of the node is not considered in CDS algorithm, the node just sends perceived environmental attributes to communication neighbors, so the number of activation nodes is fewer compared to FL-CED algorithm, but we will see that the performance of the FL-CED algorithm is better than the CDS algorithm in the detection accuracy and message exchange capacity.

**Figure 9:**
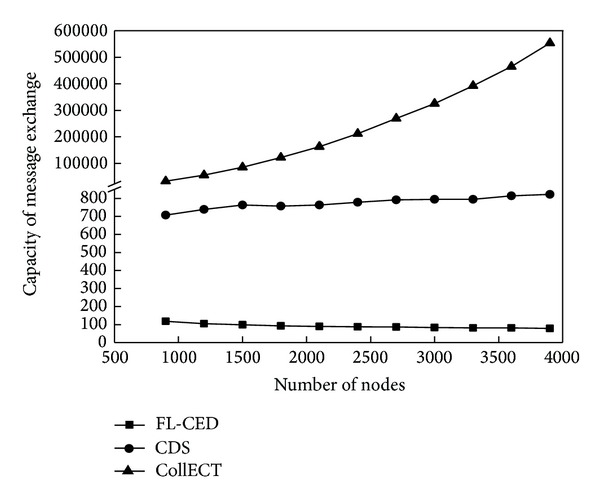
Capacity of message exchange.

Figure 9 reflects the comparison of message exchange capacity of three algorithms. The figure shows that CollECT algorithm requires a large amount of data exchange when event occurs. Sensor nodes in FL-CED algorithm only need to send the perceived environmental attributes to the selected local decision node; thus, this one-way transmission mechanism reduces the message exchange capacity further compared to the way of broadcasting adopted in CDS algorithm.

Document [35] defines the accurate rate and out-event false rate to assess the accuracy of the algorithm. Set *S*_evt_ as the set of active nodes in the area of the event, in FL-CED algorithm, *S*_evt_ is the set of the local decision node in the area of the event, *S*_urg_ is a collection of nodes that indicates an event occurs, and *N* is the total number of nodes.

*Accurate Rate*. Accurate rate is the ratio of the number of nodes that indicates an event occurs and the total number of nodes in the area of event, noted as *A*(*S*_urg_), *A*(*S*_urg_) = |*S*_urg_∩*S*_evt_|/|*S*_evt_|. Out - the Event false rate: the ratio of the number of nodes indicating an event occurs out of the event area and the total number of nodes out of the event area, noted as *E*_*o*_(*S*_urg_), *E*_*o*_(*S*_urg_) = (|*S*_urg_| − |*S*_urg_∩*S*_evt_|)/(*N* − |*S*_evt_|).

**Figure 10:**
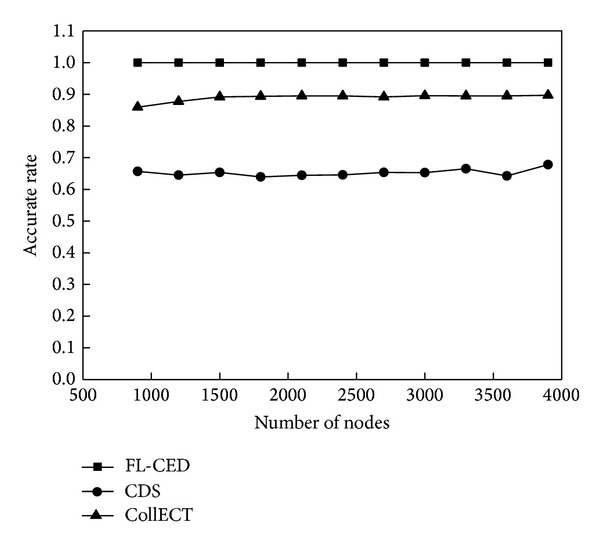
Accurate rate.

**Figure 11:**
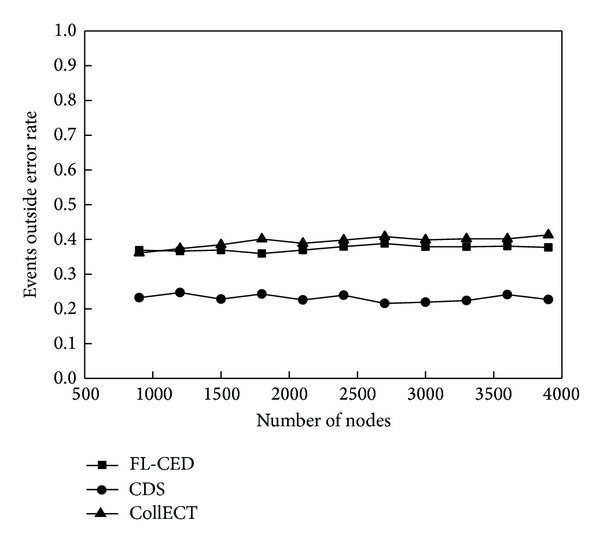
Events outside the error rate.

Figure 10 reflects accuracy of three algorithms; from the figure, the accuracy of FL-CED algorithm is close to 100%; this is due to that the perception node in the event region is able to correctly perceive events and send it to local decision node for judging. In CollECT algorithm, it is not necessarily that nodes perceiving all properties of the Composite event in network topology constituted of the node and its neighbor nodes exist; therefore, judgment that nodes make has mistakes. The accuracy of CDS algorithm is not as good as FL-CED algorithm and CollECT algorithm.  Figure 11 reflects the out-event false rate of the node: the out-event false rate of CDS algorithm is low, there is little difference between the out-event false rate of FL-CED algorithm and CollECT, around 35%, the reason why the out-event false rate of the event reaches to 35% is that the area of events is larger in the experiment, and the monitor area is smaller, but this does not affect the performance comparison of the algorithm. Although the accuracy of CDS algorithm is lower, the probability of misjudging the event of the node which is in event peripheral is lower, so CDS algorithm can reflect the external events more accurately. Because we tend to attach importance to determine the situation within the event area, so overall, accuracy of FL-CED algorithm is superior to CollECT algorithm and CDS.

**Figure 12:**
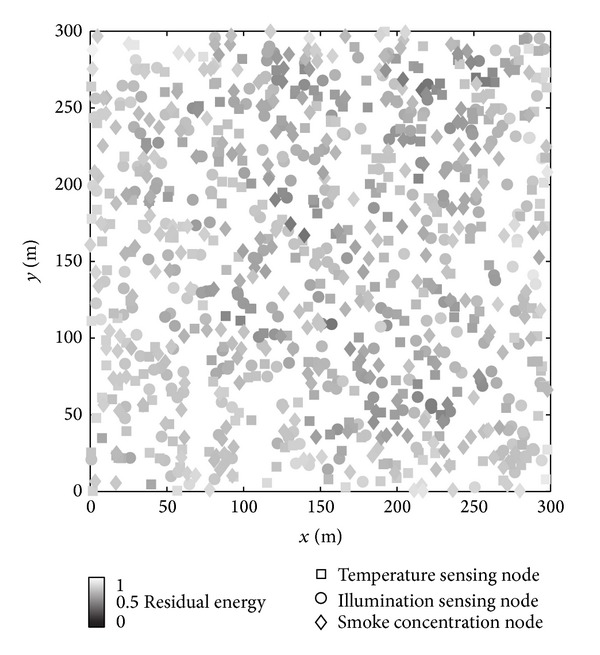
Energy distribution of DS algorithm after 250 rounds.

Since different incident detection algorithms may use different routing algorithms, so the performance of life cycle is not possible to be compared. However, from the number of active nodes and message exchange capacity, we can know that the energy consumption of FL-CED algorithm is less compared with the other two algorithms. In addition to energy consumption, energy balance is also an indicator affecting the network life cycle. Assuming that the incident happens once each round, the area is round with radius 100 m; the Figure 12 shows energy distribution after FL-CED algorithm having performed 250 rounds when *N*_0_ = 300; it can be seen from the diagram that energy consumption in the network is more balanced.

### 9.3. Fuzzy Decision of Composite Event

Data used in this study is fire testing data [35] released from the USA NIST. We will test the fire with temperature *V*_1_ and smoke concentration *V*_2_. Solution set is {*V*_1_, *V*_2_, *V*_3_}, where *V*_1_ notes sending fire event data, *V*_2_ notes sending warning data, indicating that environment is unusual, and *V*_3_ notes that no event occurs and does nothing. Constructing membership functions is based on given thresholds [37] of smoke sensor and thermal sensors Weighting matrix is *w* = {0.5,0.5}.

**Figure 13:**
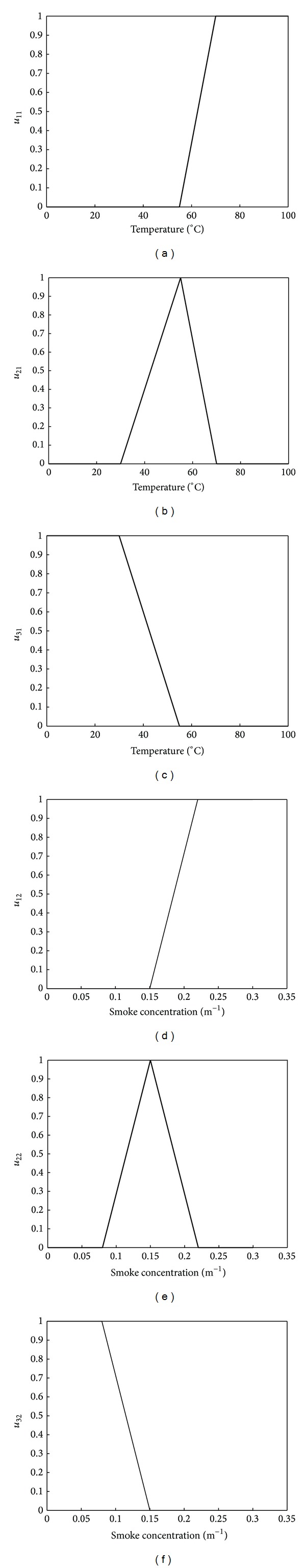
Membership functions of attributes corresponding to scheme.

**Figure 14:**
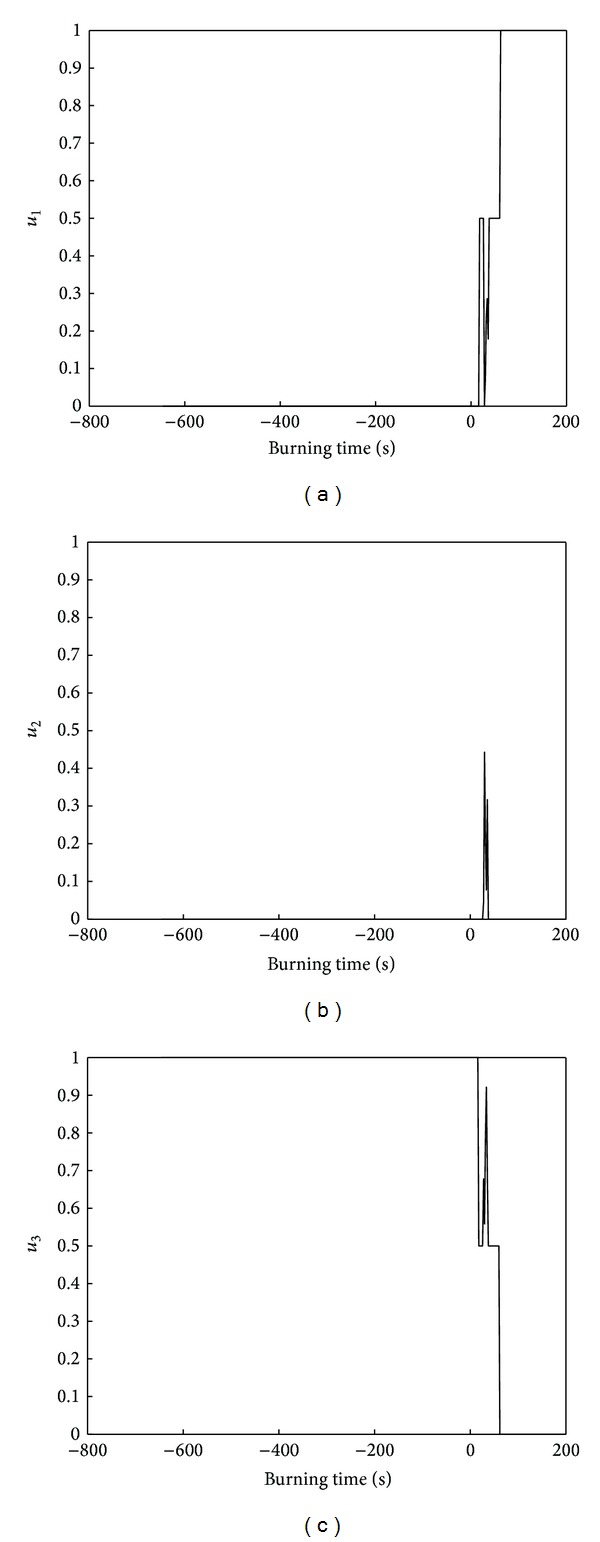
Program membership.

In Figures 13(a), 13(b), and 13(c) are membership functions *u*_11_(*x*) and *u*_21_(*x*) and *u*_31_(*x*) related to temperature, respectively. In Figures 13(d), 13(e), and 13(f) are the membership functions *u*_12_(*x*), *u*_22_(*x*) and *u*_32_(*x*) are related to smoke concentration, respectively.  Figure 14 is the membership of three schemes, and the origin is the burning time. Before catching fire, *u*_3_ is larger, so the nodes use the scheme *A*_3_, and they judge that there is no fire. At the beginning of fire, the sensor does not detect enough intensity to determine events, so *u*_3_ is larger. After a period of time, the fire expands, so the node can percept it, there is a dramatic increase in *u*_1_, *u*_3_ sharply reduce, and *u*_2_ rises a little; *u*_1_ is the largest, and the nodes take scheme *A*_1_; they judge that the fire happened. It can be seen from Figure 14 that nodes can effectively perceive the happening of the fire.

## 10. The Conclusion

Composite event is a kind of event which widely exists in the nature; the event-driven wireless sensor network is mainly used for detecting the Composite event, such as fire and intrusion detection. Due to the limitation of node itself, nodes cannot integrate all types of sensors required to detect composite events. However, the detection of Composite event requires collaboration of multiple nodes to complete. By analyzing the characteristics of the composite event, considering that different sensors have different perception, the paper has proposed a criterion of Composite event detection and designed the network topology structure algorithm CETC based on the dominating set; the algorithm will choose part of nodes in the network as local decision nodes and select a specific node as the sensing node. Local decision node makes decisions on the Composite event within its decision scope. Two-dimensional *τ*-GAS algorithm being able to screen test data correctly and decision making mechanism based on fuzzy decision are proposed. Two-dimensional *τ*-GAS algorithm can effectively filter the errors data perceived by failed node through the sliding window mechanism and clustering analysis and then calculate an estimated value of the current environment attributes. Node can determine which sensors have a permanent fault through calculating the probability of failure of the sensor. For the ambiguity of the definition of the composite event, this paper puts forward a decision mechanism based on fuzzy decision; this decision mechanism can determine whether the event has happened effectively. Compared with the decision mechanism based on fuzzy logic in the paper, this decision mechanism does not need a huge rule base. It retains the advantages of the decision-making mechanism based on fuzzy logic and can adjust to the event detection in different environment by configuring membership function matrix *I* and weight matrix *w*. The Composite event in this paper is static; it is not suitable for detecting dynamic Composite event in motion. Therefore, the tracking for mobile composite event still needs further research.

## Figures and Tables

**Algorithm 1:**
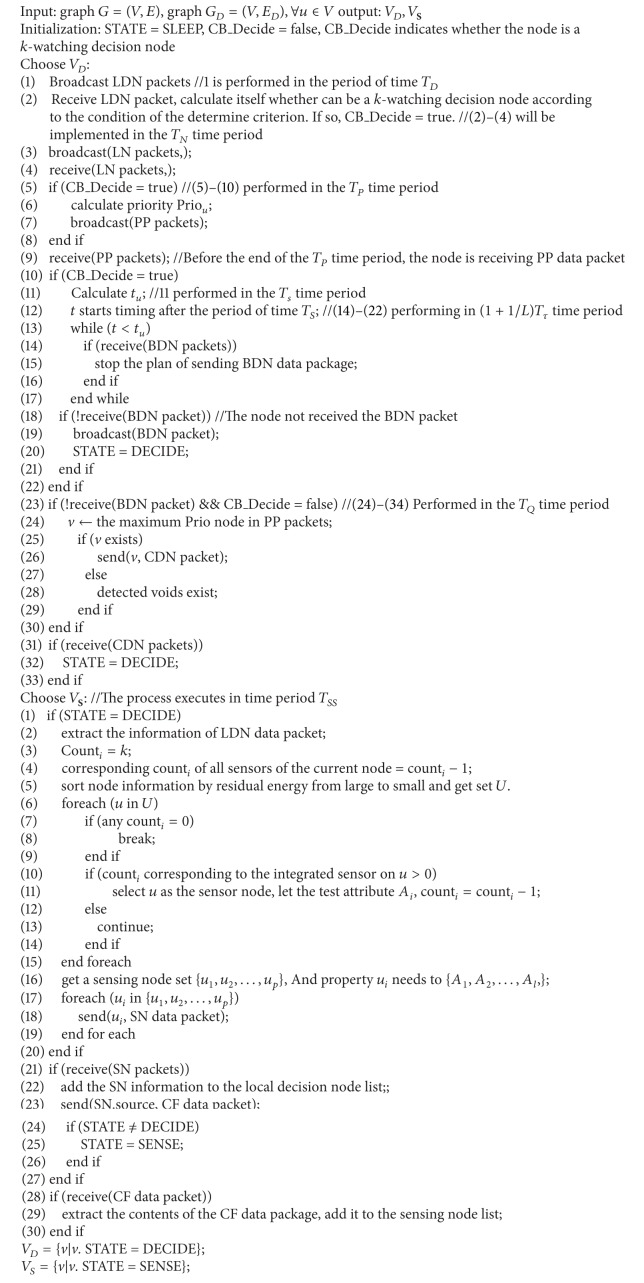
Construction algorithm of topology (CETC).

**Algorithm 2:**
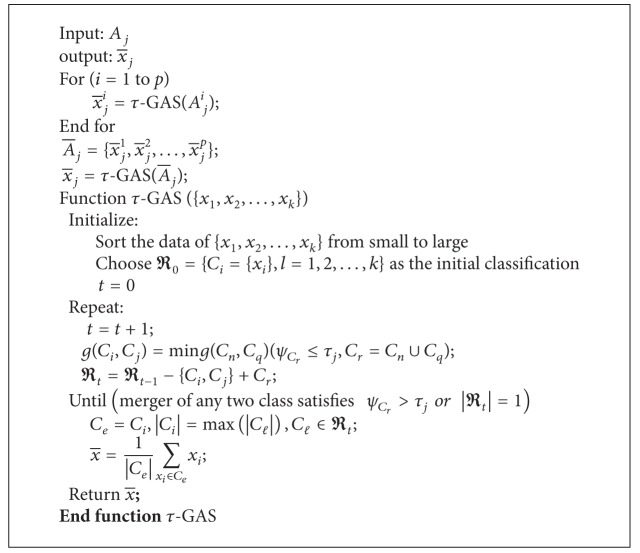
Two-dimensional *τ*-GAS algorithm.

**Table 1:** Temperature data.

*p*	*k*					
	1	2	3	4	5	6
1	53.8	55.1	55.8	54.3	78.1	55.9
2	55.1	54.9	54.1	55.5	54.1	56.4
3	55.7	81.2	54.1	55.6	21.6	55.1
4	28.9	30.7	10.1	29.7	27.8	30.5
5	54.3	53.2	55.3	54.8	55.8	53.8

**Table 2:** Settings of simulation parameter.

Parameter	Value
The area of *A*	300 × 300 m^2^
The maximum radius of *R*_*c*_	40 m
Total number of nodes, *N*	300–13000
Initial energy	1 J
*E*_elec_ (nJ*·*bit^−1^)	50
The size of data package	128 bits
The size of control package	12 bits
